# TFH cells in systemic sclerosis

**DOI:** 10.1186/s12967-021-03049-0

**Published:** 2021-08-30

**Authors:** Pauline Beurier, Laure Ricard, Deborah Eshagh, Florent Malard, Lama Siblany, Olivier Fain, Mohamad Mohty, Béatrice Gaugler, Arsène Mekinian

**Affiliations:** 1 INSERM UMRs 938, Centre de Recherche Saint-Antoine, AP-HP, Hôpital Saint-Antoine, Service de Médecine Interne and Inflammation-Immunopathology-Biotherapy Department (DHU i2B), Sorbonne Université, 75012 Paris, France; 2 Sorbonne Université, Paris, France; 3 Service D’Hématologie Clinique, AP-HP, Hôpital Saint-Antoine, 75012 Paris, France; 4 Service de Médecine Interne and Inflammation-Immunopathology-Biotherapy Department (DMU 3iD), AP-HP, Hôpital Saint-Antoine, 75012 Paris, France

**Keywords:** Systemic sclerosis, T follicular helper cells, Therapies

## Abstract

Systemic sclerosis is an autoimmune disease characterized by excessive dermal fibrosis with progression to internal organs, vascular impairment and immune dysregulation evidenced by the infiltration of inflammatory cells in affected tissues and the production of auto antibodies. While the pathogenesis remains unclear, several data highlight that T and B cells deregulation is implicated in the disease pathogenesis. Over the last decade, aberrant responses of circulating T follicular helper cells, a subset of CD4 T cells which are able to localise predominantly in the B cell follicles through a high level of chemokine receptor CXCR5 expression are described in pathogenesis of several autoimmune diseases and chronic graft-versus-host-disease. In the present review, we summarized the observed alteration of number and frequency of circulating T follicular helper cells in systemic sclerosis. We described their role in aberrant B cell activation and differentiation though interleukine-21 secretion. We also clarified T follicular helper-like cells involvement in fibrogenesis in both human and mouse model. Finally, because T follicular helper cells are involved in both fibrosis and autoimmune abnormalities in systemic sclerosis patients, we presented the different strategies could be used to target T follicular helper cells in systemic sclerosis, the therapeutic trials currently being carried out and the future perspectives from other auto-immune diseases and graft-versus-host-disease models.

## Introduction

Systemic sclerosis (SSc) is a complex autoimmune disease characterized by excessive skin fibrosis with progression to internal organs supported by activation of fibroblasts and excessive deposition of extra cellular matrix (ECM) [1]. Based on the extent of cutaneous fibrosis, two main forms of the disease have been identified, limited cutaneous SSc (lcSSc) defined by skin fibrosis restricted to distal areas and diffuse cutaneous SSc (dcSSc) associated with visceral fibrosis. In addition to the fibrotic component, major aspects of the disease include vascular involvements and dysimmunity [1, 2]. Among immunity disabilities, the homeostasis of B cells is disrupted and leads to the production of auto-antibodies and the secretion of pro fibrotic cytokines [3]. However, many studies also highlight the role of T cells and particular T helper (Th) cells in pathogenesis of SSc. While Th2 cells produce pro fibrotic cytokines including interleukin (IL)-13 or IL-4 and could participate in the activation of fibroblasts and their differentiation into myofibroblasts [4, 5], Th17 cells could promote both fibrosis and vascular impairment [6–9].

Over the last decade, aberrant responses of T follicular helper (Tfh) cells, a subset of CD4 T cells which are able to localise predominantly in the B cell follicles through a high level of chemokine receptor CXCR5 expression, are described in pathogenesis of several autoimmune diseases [10–12]. Originally described in the early 2000’s, this subpopulation was able to enhance B cell immunoglobulin production during in vitro co-culture experiments [13, 14]. Since these early reports, publications on their phenotypic characteristics and their biological functions have been intensive. Characterized by B cell lymphoma 6 (BCL-6) transcription factor expression, Tfh cells play a key role in germinal center (GC) formation, proliferation, isotypic switch and somatic hypermutation of B lymphocytes [15, 16]. Moreover, Tfh cells express co stimulatory markers including CD40L, inducible costimulator (ICOS) or programmed death (PD)-1 and produce Il-21 allowing them to participate in B cell proliferation and differentiation. In human autoimmune disease, circulating Tfh (cTfh) cells have been described. These cTfh cells present an activated phenotype and could promote B cell auto-antibody production [12].

Recently, another T cell subset sharing many common features with Tfh cells has been identified in several autoimmune diseases. These CXCR5^−^CD4^+^ICOS^+^CD40L^+^ T cells named T peripheral helper (Tph) cell are also able to help B cells and have been observed in inflamed tissue in autoimmune diseases [17]. Tph cells seem to be increased in systemic lupus erythematosus (SLE) or rheumatoid arthritis (RA) peripheral blood [18].

Here, we summarized the role of Tfh cells in SSc and potentially therapeutic way to target this subpopulation.

## T follicular helper cells in systemic sclerosis

While many investigations on the Tfh role in SLE or RA are available [10, 11], data regarding their contribution to the pathogenesis of SSc are limited.

In different animal and human models sharing immunopathological features and common fibrotic abnormalities with SSc, homeostasis and Tfh cells function are disturbed. In bronchiolitis obliterans syndrome (BOS) murine model of chronic graft versus host disease (cGVHD), Tfh cells are upregulated in the spleen and are correlated with an increase in GC B cells. Tfh cell inhibition by blocking costimulatory pathways limits GC formation and immunoglobulin production and improves lung damage [19]. During idiopathic pulmonary fibrosis, the cTfh proportion among CD4 + T cells was increased and present an activated phenotype [20]. In human cGVHD, cTfh cells seem to be decreased [21–23] but they express an activated phenotype and have a high capacity to promote B-cell immunoglobulin secretion and maturation [22].

In SSc patients, abnormalities in the number or frequency of cTfh are inconstant. While cTfh cells are increased in SSc patients compared with healthy subjects and expresses a high level of PD-1 and other activation markers including HLA-DR or ICOS in a first study [24], in the other studies, the frequency of cTfh among CD4 + T cells is similar between SSc patients and healthy subjects [25, 26]. Heterogeneity of both severity and duration of the disease and pathophysiological features could explain these observations. Indeed, SSc is a heterogeneous autoimmune disease and different clinical phenotypes have been described within the same lcSSc or dcSSc subset [27, 28]. Thus, in the first study cTfh cells were upregulated especially dcSSc and correlated with severity of skin lesions [24]. Furthermore, the analysis in the subgroup based on the cellular immunophenotype of SSc patients in the other study revealed that cTfh cells are more represented and activated in the subgroup associated with the more severe vascular damage in videocapillaroscopy [26]. Recently, a study based on homogeneous population of early dcSSc highlighted that cTfh cells are significantly increased in SSc patients than healthy subjects [29]. Moreover, loss of cTfh homeostasis has been observed [25]. According to Morita et al*.*, three subsets of cTfh are described regarding CXCR3 and CCR6 expression, cTfh1 (CXCR3 + CCR6-), cTfh2 (CXCR3-CCR6-) and cTfh17 (CXCR3-CCR6 +). Both cTfh17 and cTfh1 cells appear increased in SSc patients and associated with an increase in plasma level of IL-17F. cTfh17 secrete pro inflammatory and pro fibrotic cytokines [30, 31] and can induce B cell differentiation. Elevation of cTfh17 frequency is reported in several immune diseases or during cGVHD and contributes in pathogenesis [22, 32–34].

Tfh cells induce B cells differentiation and promote immunoglobulin secretion by IL-21 secretion. During SSc, cTfh cells impairment is associated with an imbalance of B cell subsets [25, 35]. Frequencies of both naïve B cells and plasmablasts are increased while the frequency of memory B cells is reduced. Moreover, a higher plasma level of IL-21 is found in sera from patients than in healthy subjects and is correlated with plasmablast numbers, suggesting that a dysregulation of cTfh in SSc patients could be responsible for B cells alterations. In vitro Tfh cells co-cultured with autologous B cells from SSc patients enhanced plasmablast differentiation and induced high level of immunoglobulin production [24]. IL-21R blockade reduces the Tfh cells capacity to stimulate the plasmablasts and decreases Ig secretion.

In addition to the cTfh cells and because they also have the ability to help B cells, circulating Tph (cTph) cells represent an interesting target subset. Recently, Fox et al. observed that this subset is decreased in early dcSSc patients compared to controls [29]. However, their function and the evolution of their frequency over time remain unknow in the context of SSc.

Taken together, cTfh cells are dysregulated in SSc and appears to be increased especially in dcSSc form and during the early phase of the disease. They produce high levels of IL-21 and express costimulatory signals which could support aberrant B cell activation and differentiation, responsible for immunologic abnormalities. However, although it appears that cTfh cells may be altered in SSc, we don’t currently know whether this impairment is the result of a chronic immune activation or corresponds to a clonal selection by an antigen specific.

In tissue, several studies conducted in animals and humans have described that T cells are involved in skin fibrosis generation [36, 37] and Tfh-like cells infiltrates were found in skin lesions from SSc patients [25, 38, 39]. This infiltrate have an increased frequency than healthy subjects and is positively correlated with mRSS and with a breakdown product of collagen I involved in ECM synthesis [38]. In vitro, co-culture of normal human dermal fibroblasts with differentiated Tfh-like cells drives myofibroblast differentiation suggesting an implication of Tfh-like cells in fibrogenesis [38]. Moreover, several studies suggest that Il-21, one of the main cytokine produced by Tfh cells [15], may have a pro-fibrotic effect in diverse autoimmune diseases. Indeed, Il-21 could promote in vitro both the differentiation and proliferation of fibroblast-like synoviocytes in RA [40] but also could induce their secretion of pro-fibrotic markers and matrix metalloproteinases in RA or inflammatory bowel disease [40–42]. In sclerodermatous cGVHD mice models, inhibition of Tfh-like cells using anti-ICOS depleting monoclonal antibody (Mab) improves cGVHD manifestations and decreases both IL-21 and IL-21 receptor expression. Furthermore, IL-21 neutralization leads to improvement of skin damage and inhibits Tfh-like cells and profibrotic marker gene expression [38].

However, while it appears that Tfh-like cells are present in fibrotic lesions of SSc patients, limited data are available on the frequency of this subset in pathological tissue. Furthermore, other subsets of T cell such as cytotoxic CD4 + T cells or other pro fibrotic immune cells have also been identified in the skin lesions and may also contribute to fibrogenesis [7, 39, 43, 44]. Currently, the exact interaction between Tfh-like cells and theses others cells in tissues is unknow. Moreover, whereas Tfh-like cells may promote the differentiation of fibroblasts in vitro, the role of the fibroblasts in both Tfh cell differentiation and clonal selection of this subset remains undetermined [45]. Thus, further analysis of the significance of the presence of Tfh-like cells in skin lesions are needed.

Overall, Tfh cells may be involved in both immunological and fibrotic abnormalities in SSc. Although a better comprehension of the link between abnormal Tfh cell increase, autoreactive B cell expansion and fibroblast activation is necessary, targeting Tfh cells could potentially become a promising new therapeutic avenue in this particularly complex autoimmune disease.

## Targeting TFH in systemic sclerosis: Perspectives from other auto-immune diseases and GVHD models

**Fig. 1:**
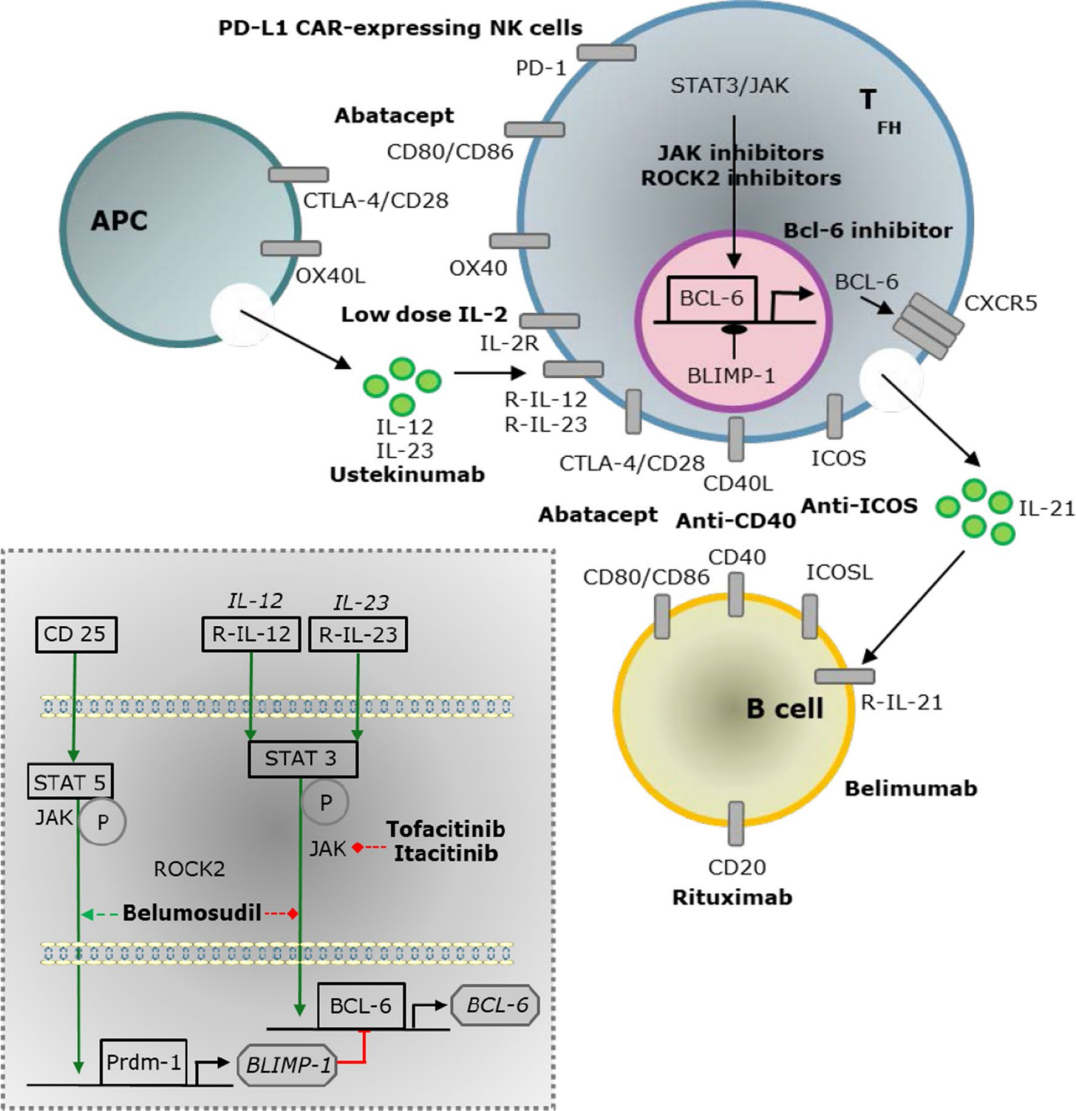
Different strategies could be used to inhibit Tfh cells during SSc. APC: antigen presenting cell, BCL-6: B cell lymphoma 6, BLIMP-1: B lymphocyte-induced maturation protein 1, CAR: chimeric antigen receptor, CD: cluster differentiation, CTLA-4: cytotoxic T-lymphocyte-associated protein 4, CXCR5: C-X-C motif chemokine receptor 5, Il: interleukin, ICOS: inducible T-cell costimulator, JAK: janus kinase, NK: natural killer, PD-1: programme death, PD-L1: programmed death ligand, TFH: T follicular helper, Prdm-1: PR domain zinc finger protein 1, STAT: signal transducer and activator of transcription 1, TGF-β: transforming growth factor-β

Because Tfh cells are involved in both fibrosis and autoimmune abnormalities in SSc patients, targeting Tfh cells represents an interesting therapeutic pathway. Different strategies can be used to target Tfh cells in systemic sclerosis (Fig. 1). Some treatments potentially targeting Tfh cell signalling or costimulatory pathways are in clinical trials for this disease (Table 1).

**Table 1:** Targeting Tfh cell signalling and costimulatory pathways: Current clinical trials

Target	Drug	NCT number (status)	Design	Primary end-point
*JAK/STAT signalling pathway*	Belumosudil [55, 56]	03919799 (recruiting)	Phase II Randomized, Double-Blind, Placebo-controlled study	Combined Response Index in Diffuse Cutaneous Systemic Sclerosis (CRISS) at Week 24
		04680975 (recruiting)	Phase II, Open label, Multicenter Study	CRISS at Week 24
	Itacitinib [59]	04789850 (Not yet recruiting)	Phase II, Randomized, Quadruple-Blind, Placebo-Controlled Study	Change in modified Rodnan skin score (mRSS) at 360 days
*B cells*	Belimumab and rituximab combination therapy (75)	03844061 (recruiting)	Phase II, Randomized, Double-Blind, Placebo-Controlled Study	Change in the American College of Rheumatology (ACR) CRISS at 12 months and the proportion of participants who experience at least one Grade 3 or higher adverse event at or before 12 months

Tfh cells differentiation and activation depend on multifactorial processes and are regulated by several signalling pathways. Among them, the signal transducer and activator of transcription (STAT)3/janus kinase (JAK)2 signalling pathway is overexpressed in SSc [46]. Activated by several cytokines and growth factors, STAT3 is involved in skin fibrosis in both humans and bleomycin induced mouse model and also in Tfh cell differentiation and contributes to BCL-6 expression [16, 47, 48].

Rho-associated kinase 2 (ROCK2) is an isoenzyme which is also involved in STAT3 phosphorylation, the STAT3/JAK signalling pathway, and BCL-6 expression in human T cells [49]. In healthy subjects, inhibition of ROCK2 by belumosudil KD025, an oral specific ROCK2 inhibitor, induced down regulation of STAT3 phosphorylation and its transcriptional activity in ex vivo activated T cells [50, 51]. Inhibition of ROCK2 also leads to decrease IL-17 and IL-21 levels in sera [51, 52]. In a murine multi-organ system cGVHD with BOS, ROCK2 inhibitor decreases the frequency of Tfh cells in animal spleens [53]. In sclerodermatous cGVHD mice, belumosudil reduces STAT3p expression and leads to skin improvement [53]. Targeting ROCK2 shows promise for SSc patients. A recent phase 2 open-label, randomized, multicenter study using belumosudil in patients with cGVHD who received previous treatments showed effective responses with a well-tolerated profile [54]. In addition, two trials (NCT 03919799 and 04680975) assessing efficacy of belumosudil in diffuse cutaneous SSc patients are currently being conducted [55, 56].

Moreover, in recent years, several studies have focused on JAK inhibition for the treatment of both GVHD and auto-immune diseases [57]. During in vitro experiments using peripheral blood mononuclear cells (PBMC) from SSc patients, JAK inhibitor reduces STAT phosphorylation [46], suggesting that its use should to be promising for SSc treatment. Indeed, tofacitinid, a pan inhibitor of JAK has been evaluated in early diffuse cutaneous SSc and found to be well tolerated [58]. Although another phase II trial of the JAK1 inhibitor itacitinib was recently been opened in adult SSc patients (NCT 04789850) [59], further studies could be conducted to assess its clinical benefits.

Several other positive or negative costimulatory signals participate in Tfh cell differentiation and activation. Among them, CD40L and ICOS, and its binding partners CD40 and ICOS-L expressed by B cells or dendritic cells (DCs) are involved in the pathogenesis of SSc [60, 61] and strongly expressed by cTfh in SSc patients [24, 25]. In animal models, blocking ICOS or CD40L reduces the frequency of Tfh [19]. Although there are currently no trials of therapy targeting CD40L or ICOS in SSc patients, a promising study in patients with rheumatoid arthritis using of an anti-CD40 antagonist Mab leads to a decrease in activated B-cells and autoantibody production [62].

While the interaction between CD28 and CD80/CD86 promotes T cell activation, that of CTLA-4 or abatacept, a CTLA-4 Ig protein fusion, with CD80/CD86 inhibits the immune response. In bleomycin-induced dermal fibrosis and sclerodermatous cGVHD [63], abatacept prevents dermal fibrosis in the early stage of the disease and may reduce established skin fibrosis. Moreover, in other mouse models mimicking SSc organ damage [64], abatacept improves lung, liver and gastrointestinal tract injuries. In both studies, a reduction in the infiltration of T cell into lesions tissues is observed. These data are supported by a phase 2 placebo-controlled study assessing the impact of abatacept in patients with multiple sclerosis. In this trial, phenotypic analysis of cell subpopulation after treatment highlights a reduction in the frequency of both cTfh cells and plasmablasts [65]. A pilot study [66] and a multicenter double-blind, randomized placebo-controlled phase 2 trial in early diffuse cutaneous SSc [67] found that abatacept is a well-tolerated treatment. Although an improvement in composite clinical score has been released, further trials will be needed to assess the clinical efficacy of abatacept and to confirm its Tfh-targeting action in SSc.

The relationship between Tfh cells and B cells plays an important role not only for the differentiation and activation of B cells, but also in the final stage of differentiation of Tfh cells [15, 68]. B cell depletion-based therapies using antiCD20 or anti B cell activating factor (BAFF) have been reported in recent years. In a prospective, multicenter phase 2 trial in cGVHD patients, rituximab, a chimeric Mab targeting CD20 induced a reduction on cTfh cells [69]. The same results are released in patient with immune thrombocytopenia [70]. In SSc patients, the use of rituximab results in a decrease in CD4 + CD40L + T cells in blood of patients compared to controls [71]. A recent meta-analysis suggests that rituximab may improve skin lesions and stabilize lung impairment in SSc [72]. Moreover, BAFF is upregulated in serum from SSc patients [25, 73] and associated with skin and lung damage in bleomycin-induced scleroderma models [74]. Inhibition of BAFF in this animal model induced an improvement in the fibrotic injury in tissues [74]. A randomized, double blind placebo-controlled study on the combination of belimumab and rituximab for the treatment of diffuse cutaneous SSc is currently being carried out (NCT 03844061) [75].

Although targeting costimulatory or signalling pathways can lead to the inhibition of Tfh cells, these strategies remain unspecific. Other approaches could be considered to better control Tfh cells specifically. Thus, while the use of a direct inhibitor of Bcl-6 in nonsclerodermatous cGVHD seems promising, its efficacy in skin fibrosis remains uncertain [76]. Further studies are needed to better characterize the efficacy of this approach in SSc. Furthermore, whereas IL-21 blockade can decrease plasmablasts differentiation in vitro or in animal models, trials using IL-21 or IL-21R blockade in humans are lacking.

Another interesting strategy would be to target the polarization of Tfh cell. During SSc, the DCs which play an important regulatory role in antigen presentation and polarization of naive T cells, are altered and produce high levels of IL-12, a potential cytokine involved in Tfh cell differentiation [15, 77–79]. Furthermore, others antigen presenting cells (APCs) and in particular SlanMo, a subset of non-classical monocytes known to be a major source of IL-12 and IL-23 was impaired in several autoimmune diseases [80]. A recent study using the Mab ustekinumab directed against the shared p40 subunit of IL-12 and IL-23 in crohn’s disease patients showed that this treatment leads to a decrease in Tfh cell differentiation in vitro [81]. Unfortunately, limited data are currently available on the interaction between APCs and Tfh cells in the context of SSc. However, modulating the polarization of naïve T cells appears to be a promising pathway for the development of future therapies.

Imbalance between effector and regulatory cells supported by Tfh dysregulation is one of the key mechanisms leading to a breakdown in immune tolerance in auto-immune diseases. Since IL-2 could promote the maintenance of regulatory T cells (Treg), some studies based on IL-2 therapy for the treatment of auto-immune disease or cGVHD have been published [82–84]. In SLE patients, the lack of IL-2 and the imbalance between Tfh and Treg cells could be restored after low dose IL-2 treatment [85, 86]. Indeed, IL-2 could inhibit Tfh cells depending on the intensity of signal. IL-2-induced phosphorylation and activation of STAT5 increases B lymphocyte induced maturation protein (BLIMP)-1 expression and therefore inhibits the BCL-6 antagonist factor [87, 88]. Furthermore, a study suggests that IL-2 may promote in vitro the conversion of Tfh to T follicular regulatory (Tfr), a subset of CD4 + helper T cells that express both FOXP3 and BCL6 factors and are able to repress Tfh and GC in B follicles [89]. Moreover, data on Treg cells during SSc are inconsistent [90]. While several studies describe a decrease in circulating Treg in SSc patients, others shown an increase in this subset, especially in the early stages of the disease. However, data on the involvement of Tfr cells in SSc are lacking. Further characterizations of the Treg and Tfh/Tfr imbalance are necessary to evaluate the potential benefit of low dose IL-2 therapy.

Tfh cells express higher levels of PD-1. Based on this observation, Reinhardt et al*.* have engineered a PD-L (programme death ligand)1 based chimeric antigen receptor (CAR) natural killer (NK)-cell that targets PD-1-expressing cells to eliminate Tfh [91]. Co-culture experiments of CD4 T cells sorted from healthy human tonsil with PD-L1 CAR NK-cell induced a reduction of Tfh cells and a loss of viability in remaining cells. Moreover, in co-culture experiments with human tonsillar lymphocytes enriched for Tfh cells and memory B cells, PD-L1 CAR NK-cell induced a decrease in prevalence of plasmablasts and IgG. Finally, in mouse model of lupus-like disease, PD-L1 CAR NK-cells decreased CD4 T cells [91]. Taken together, these data suggest than PD-L1 CAR NK-cell can induce a reduction in the B cell differentiation into plasmablast though decrease in Tfh cells. In SSc patients, cTfh cells express high level of PD-1 [24] and inhibition of these cells by targeting PD-1 with CAR NK technology represent an interesting treatment pathway.

## Future perspective

Although we have observed that Tfh cells are implicated in pathogenesis of SSc and constitute an interesting target for current and future treatments, the exact interaction between Tfh cells and other major protagonists in SSc remains partially understood. Elaboration of the link between anormal Tfh cells increase and autoreactive B cells expansion, fibroblasts activation and profibrotic mediator production could provide a better understanding of the pathophysiology of the disease.

One of the major questions in SSc is the mechanism underlying the altered Tfh cell homeostasis in both peripheral blood and pathological tissue. Although this is accompanied by an imbalance between B cell subpopulations, it is currently unclear whether Tfh cell responses are the result of clonal selection driven by a specific autoantigen or the consequence of persistent immune activation. A recent study conducted by Servaas and al. using high-throughput sequencing of TCRβ chains in SSc highlighted persistence of the TCRβ repertoire for CD4 + and CD8 + T cells in the same patient over time [92]. Using the clustering analysis “grouping of lymphocyte interactions by paratope Hotspot 2” the authors showed the presence of groups of T cells that potentially responded to the same antigen, suggesting a clonal selection of T cell by a specific antigen in SSc [92]. Further characterization of the TCR repertoire of Tfh cell would be necessary to better understand the mechanisms underlying the expansion of this subset.

Thus, a better understanding on the origin of Tfh cells dysregulation and the exact consequence on immune and fibrotic processes could provide a better overview of the link between cutaneous and organ fibrosis and dysimmunity during SSc and could open up avenues to define new therapeutic strategies to modulate the immune system.

## Data Availability

Not applicable.
